# Development and Validation of AI Help-Seeking Behavior Scale Among Undergraduate University Students

**DOI:** 10.3390/ejihpe16070090

**Published:** 2026-06-29

**Authors:** Othman A. Alfuqaha, Rasha M. Abdelrahman, Kyle Msall

**Affiliations:** 1 Psychology Department, Faculty of Humanities and Sciences, Ajman University, Ajman 346, United Arab Emirates; r.abdelrahman@ajman.ac.ae (R.M.A.); k.msall@ajman.ac.ae (K.M.); 2 National Center for Examinations and Educational Evaluation (NCEEE), Al Abageyah, El Mokattam, Cairo 11341, Egypt

**Keywords:** Ajman University, artificial intelligence, help-seeking behavior, university students, validation

## Abstract

(1) Background: Artificial intelligence tools have become integrated into undergraduate students from academic assignments to seek help with psychological concerns, particularly during the crises period. Scales measuring Artificial Intelligence-help-seeking behavior (AI-HSB) are still limited. This study aims to develop a new bilingual scale (Arabic and English) to assess AI-HSB by providing a reliable and useful tool for researchers worldwide. (2) Methods: We conducted a methodological cross-sectional design among 416 undergraduate students in United Arab Emirates (AUE) between the period of 1 October 2025 and 10 December 2025, using an online Google Form. The development, translation, validation, and reliability processes were conducted for the AI-HSB scale. (3) Results: It has been found that 13 items (two factors) are strong indications of factorial validity, reliability, and construct validity of AI-HSB scale. The two factors explained about 58% of the total variance. The confirmatory factor analysis confirmed the two-factor structure with all items loading above recommended thresholds and the goodness-of-fit indices of AI-HSB all exceeded 0.90. (4) Conclusions: The AI-HSB is a valid and reliable tool for assessing AI-based psychological help-seeking behavior among university students in the UAE. This scale will allow universities, counselors, and policymakers to use a well-validated scale to measure the extent to which students are using AI for psychological coping.

## 1. Introduction

Artificial intelligence (AI) technologies have become integrated into contemporary higher education. In recent years, university students have gradually turned to digital systems for academic assistance, learning support, and coping psychologically. Advances in emotionally intelligent AI systems, conversational agents, and adaptive tutoring platforms have enabled rapid, personalized interactions that simulate aspects of human support. At the same time, researchers have drawn attention to the ethical challenges of deploying Al in sensitive contexts, including concerns about privacy protection, emotional reliance, and human oversight in mental-health support (Albikawi et al., 2025; Saeidnia et al., 2024).

These developments position AI not merely as an instructional technology but as a potential help-seeking agent in students’ academic and emotional lives. University students constitute an important population for understanding AI-mediated help-seeking behavior. The transition to university is accompanied by heightened academic pressure, financial strain, lower physical activities, and psychosocial challenges, contributing to elevated prevalence rates of depression, anxiety, and stress across global contexts (Yasin et al., 2026; Limone & Toto, 2022). Thus, AI tools, such as ChatGPT and chatbots, have become part of university students’ daily lives and are used to seek help with academic tasks, psychological concerns, and even social interactions (Vieriu & Petrea, 2025). Several previous studies found that 85% of undergraduate students had used ChatGPT for academic assignments, emotional purposes, and coping strategies to tackle the symptoms of stress, depression, and anxiety (Sallam et al., 2024; O. A. Alfuqaha et al., 2026). Despite the increasing number of students using AI tools, the debates still regarding the trust of AI tools and how they rely on AI applications for psychological support (O. A. Alfuqaha et al., 2026).

The HSB can be defined as part of self-regulated learning and adaptive coping that may refer to students’ willingness to seek help from teachers, peers, or professionals when struggling with academic or emotional problems (Wilson et al., 2005b). Researchers often distinguish between instrumental help-seeking, which supports learning, and maladaptive forms of help-seeking that involve avoidance or dependency (Picco et al., 2016). The rise in AI tools introduces new affordances, immediate access, and rapid responsiveness-that may influence students’ help-seeking decisions. As emotionally intelligent systems become more capable of detecting affective states and adapting responses, they may enhance perceived support and engagement, yet they also raise concerns about psychological, emotional dependence and the potential displacement of human support (Wu, 2024).

Seeking help from formal resources, such as psychologists and psychiatrists, when needed, is paramount. But nowadays, AI tools have introduced a new pathway to seek psychological help, emotional support, and trust on AI tools to solve psychological problems such as anxiety, stress, and other mental issues. The researchers theoretically proposed that the AI-HSB is expected to have two directions: positive and negative. The positive direction refers to the technology acceptance model (TAM), which highlights that using technological tools can be useful, accessible, and beneficial (Venkatesh & Davis, 1996; O. Alfuqaha et al., 2022). In contrast, the other direction is related to the barriers to help-seeking model. It refers to barriers, privacy concerns, and avoiding seeking support from AI tools instead of human interactions (Velasco et al., 2020). Thus, AI-HSB is expected to represent facilitating belief and perceived barriers that can limit its use.

Even with the rapid growth of research on AI in psychology and mental health, there are still few validated measures that capture AI-specific help-seeking for how much individuals rely on AI, perceived usefulness, attitudes toward AI mental health tools, or their concern about using AI tools. Most studies have previously focused instead on technology acceptance, attitudes toward Al, or general orientations toward psychological help-seeking (AlMaskari et al., 2025; Alam et al., 2025; Swidan et al., 2025). Although several recent studies have adapted traditional help-seeking scales to assess reliance on psychological concerns, psychometric validation of AI-specific help-seeking constructs, and reliance on AI for psychological guidance remain limited (Picco et al., 2016; Wilson et al., 2005a). The absence of well-developed and validated instruments limits researchers’ ability to examine individual differences in reliance on AI-HSB, assess potential risks of overdependence, and guide institutional policies on responsible Al use in higher education. Thus, to address this gap, the present study develops and validates a new AI-HSB scale in the English and Arabic languages for undergraduate students. The researchers conducted exploratory factor analysis (EFA), followed by confirmatory factor analysis (CFA) to examine factor structure of the new AI-HSB scale.

## 2. Materials and Methods

### 2.1. Study Design

We used a methodological cross-sectional design to develop a new tool for measuring AI-HSB among undergraduate university students.

### 2.2. Settings and Procedures

The researchers targeted university students as they use AI tools frequently for homework/assignments and other reasons. Besides that, university students have higher levels of psychological distress, such as burnout (O. A. Alfuqaha et al., 2025). Populations other than this specific group were excluded. The study sample was recruited at Ajman University, United Arab Emirates (UAE), because it was readily accessible to the researchers. The inclusion criteria comprised all university students pursuing a bachelor’s degree across different schools and academic years, aged 18 years and above, and were able to read and write in the Arabic and English languages. The exclusion criteria included postgraduate students because they are assumed to have greater professional experience and higher autonomy. Data collection was conducted between 1 October 2025, and 10 December 2025, using an online Google Form (Google LLC, Mountain View, CA, USA). The form was officially distributed via a university email and through WhatsApp university student groups. Participation was completely voluntary and no academic credits or incentives were provided for the students. The Google Form was presented in both languages since we validated bilingual development of the AI-HSB scale. During that period, the total number of respondents was 416, which was considered sufficient, as scale development studies recommend at least 10 participants per item (Morgado et al., 2017). General information was requested from each university student in the UAE, including gender, marital status, age, academic year, and major (humanities and sciences, business, medical, information technology, architecture/art, and engineering). The form requires approximately 3 min to complete.

### 2.3. Item Generation

The generations of AI-HSB items rely on three sources: existing literature, previously published studies, and expert feedback in the relevant field. The existing literature was based on reviewing the background and literature on HSB, including theories and new models such as TAM and the barriers to help-seeking models, new books about AI, online mental health support, and AI-based psychological support (Chigbu et al., 2023). Second, the researchers referred to several previous studies to develop a new scale measuring AI-HSB among university students. In this regard, the researchers referred to previous scales that measure tradition professional psychological help (Picco et al., 2016), general help-seeking behavior (Wilson et al., 2005a), and orientation to seeking professional help (Fischer & Turner, 1970). Third, the researchers requested 8 arbitrators specialized in psychology, counseling, AI, and mental health fields to establish new items based on their expertise in their fields. After intensive discussion and consensus between the researchers based on the three resources, the initial number of items emerge was 13 in the English language measuring reliance on AI-HSB for psychological/emotional problems, resolution of psychological distress, and concern toward using AI for seeking help. However, the researchers use four-point Likert-type scale from strongly agree to strongly disagree. For descriptive interpretation purposes, we relied on mean scores divided into three categories, which are: low reliance on AI-HSB (1.00 to 1.99), moderate reliance on AI-HSB (2.00 to 2.99), and high reliance on AI-HSB (3.00 to 4.00).

### 2.4. Translation Phase

This process was initiated after agreeing on 13 items that measure AI-HSB in the English language. To translate these items into the Arabic language, the researchers requested a professional expert in English–Arabic translation to translate them. After that, the researchers requested another professional expert in Arabic–English to translate the items from the Arabic language back into English (Grover et al., 2025). At least 90% agreement on each item will be accepted.

### 2.5. Face and Content Validity

Assuring that AI-HSB items are clear, appropriate, and cover the conceptual framework, the researchers conducted both face and content validity. Face validity relies on asking pilot participants, consisting of 40 participants, to fill in both language versions of the AI-HSB regarding clarity, appropriateness of items, and ease of understanding. Regarding content validity, the researchers asked a total of 8 arbitrators specialized in counseling, psychology, and AI-related mental health to provide their feedback on the AI-HSB in both languages using a three-point Likert-type scale (appropriate, belonging, culturally adapted). The Content Validity Ratio (CVR) was calculated based on Lawshe’s CVR Table; a minimum value of 0.75 was required (O. A. Alfuqaha et al., 2022). The Content Validity Index (CVI) was calculated using a four-point Likert-type scale, ranging from not relevant to strongly relevant; thus, a minimum value of 0.78 was required (Cocchi et al., 2023).

### 2.6. Explanatory Factor Analysis

To perform cross-validation, the researchers split the whole data set (416 participants) randomly into two halves, one for EFA and one for CFA (de Rooij & Weeda, 2020). Thus, the researchers first conducted an EFA model to examine the factor structure of AI-HSB, and then, the researchers re-evaluated this model using CFA on a separate data set. By a random procedure, the total number of datasets on EFA was 219, and for CFA, it was 197 participants. The researchers conducted the following analysis criteria for EFA: factor loading (Varimax rotation) above 0.40, Kaiser–Meyer–Olkin (KMO) > 0.6, Bartlett’s Test of Sphericity must be significant, and eigenvalues more than 1 with a scree plot were considered as factors (Sigudla & Maritz, 2023). The percentage of variance explained and the Varimax rotation method, with factor loadings equal to or above 0.40, were considered sufficient (Tavakol & Wetzel, 2020).

### 2.7. Confirmatory Factor Analysis

The researchers conducted CFA to confirm the factor structure of AI-HSB on a different dataset with a total number of 197 university students. The researchers conducted the following analysis criteria for CFA: items loading on each factor of AI-HSB were required to be above 0.40 (Tavakol & Wetzel, 2020). Model fit indices included Chi-square (<3), goodness-of-fit index (GFI), Comparative Fit Index (CFI), and Incremental Fit Index (IFI), Tucker–Lewis Index (TLI); all of these values should exceed 0.90 (Stone, 2021). The Root Mean Square Error of Approximation (RMSEA) was required to be less than 0.05, suggesting an acceptable model fit (Shi et al., 2019).

### 2.8. Convergent and Discriminant Validity

The purpose of conducting convergent validity is to examine the association between constructs and their items. It was assessed using average variance extracted (AVE > 0.50) (Cheung et al., 2024). While the discriminant validity aimed to measure the construct distinct from other constructs that can be assessed through the squared root of AVE, which should exceed inter-factor loading (Cheung et al., 2024).

### 2.9. Reliability

The researchers checked the reliability among CFA datasets by conducting Cronbach’s alpha (alpha > 0.60) and composite reliability (CR > 0.70) based on previous recommendations that suggest reliability among CFA, not EFA datasets (Hair et al., 2019).

### 2.10. Data Analysis

Both SPSS version 30.0 (IBM Corp., Armonk, NY, USA) and AMOS program version 30.0 (IBM Corp., Armonk, NY, USA) were conducted to measure EFA and CFA of the AI-HSB scale, respectively. We split the data set into two halves, one for EFA (219 participants) and one for CFA (197 participants). For the EFA, we conducted varimax rotation, KMO, Bartlett’s Test of Sphericity, and eigenvalues. For CFA, we conducted Chi-square, GFI, CFI, TLI, IFI, and RMSEA. *p*-value < 0.05.

### 2.11. Ethical Approval

Ajman University in UAE approved this study (Approval No.: H-F-H-23-Sep). The researchers stated on the Google Form that if the students consent to be part of this study, they should click Yes before proceeding; otherwise, they will be excluded. All necessary information and details were provided to students, including the purpose, anonymity, and confidentiality.

## 3. Results

### 3.1. Demographic Information

The selected university, which is located in Ajman-UAE, had different nationalities and cultural backgrounds from various countries. Regarding the nationality of participants, they were UAE nationals and expatriate residents. However, the total number of our participants in this study was 416 university students, most of whom were single, female, and had an age between 18 and 20 years old. Nearly half of the participants were in the first academic year (49.3%). Students enrolled in humanities and sciences are the majority of respondents compared to other majors, with a percentage of 26.4%; for more details, see Table 1.

### 3.2. Item Generation

Based on reviewing the existing literature, published studies, and expert feedback, a total of 13 items emerged in the English language. Several items were modified based on wording and clarity after intensive revision from the researchers, and, thus, no items were omitted from the 13 initiated items. All items were related to AI-HSB, measuring mental health-related help-seeking and positive attitudes to reliance on AI to support mental health, and resolve psychological distress.

### 3.3. Translation Phase

Translation experts provided a robust translation from English to the Arabic language, and other experts did the back translation. The final agreements on each item exceeded the threshold. Thus, the AI-HSB scale is translated into both languages. For more details, please refers to Supplementary Materials Table S1.

### 3.4. Face and Content Validity

Pilot participants were asked to give their feedback on the AI-HSB scale after the translation process in both languages. They suggested amendments to some items based on vocabulary, clarity, and appropriateness. The researchers considered these recommendations and amended some vocabulary. After that, the researchers sent the AI-HSB scale to eight arbitrators to provide their important feedback on the AI-HSB scale; hence, the CVR and CVI were 0.88 and 0.91, respectively.

### 3.5. EFA

**Figure 1:**
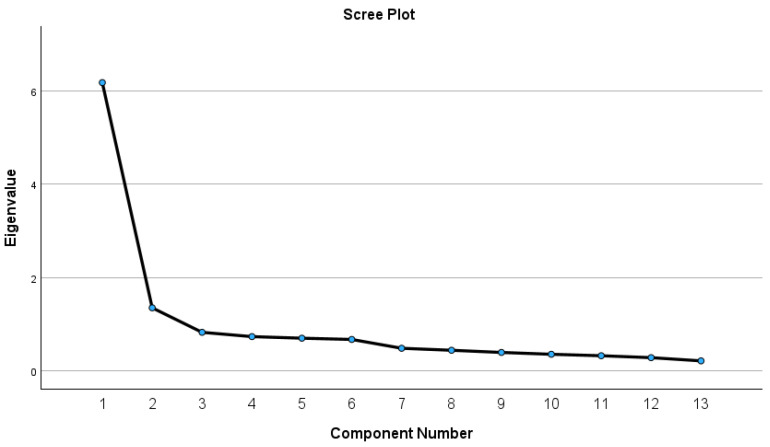
Scree plot of AI-HSB scale (13 items).

The total number of datasets for EFA was 219 participants. The Varimax rotation method revealed that all items of the AI-HSB scale had factor loadings greater than 0.40. The KMO value was 0.93, and Bartlett’s test of sphericity was significant (Chi-square (χ^2^) = 1379.186, df = 78, *p* < 0.001). Furthermore, two constructs were loaded (Figure 1). The first construct contained 10 items with an eigenvalue of 6.181. The researchers labeled the first construct with a positive attitude toward AI-HSB. The second construct covered three items (items 4, 9, and 10) with an eigenvalue of 1.354. It was labeled with concern about using AI-HSB. These constructs explained a total cumulative variance of 57.959% (Table 2). These values indicate that the AI-HSB achieves the criterion levels of EFA.

### 3.6. CFA

**Figure 2:**
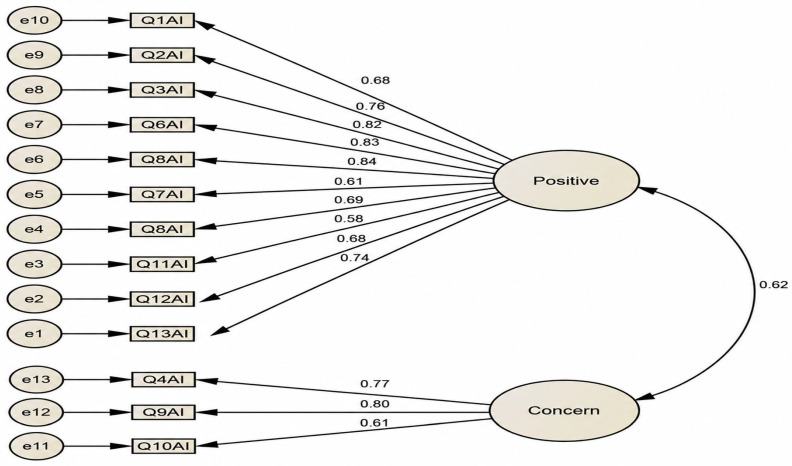
CFA model of the AI-HSB scale (*n* = 197).

The researchers conducted the CFA on a sample of 197 participants. The model consisted of two constructs (positive attitude and concern about using AI), with standardized factor loadings appearing in Figure 2. All items with their constructs exceeding the acceptable threshold of 0.40. The intercorrelation between the two constructs was 0.62. Moreover, the overall model was statistically significant (χ^2^ = 1166.29, df = 78, *p* < 0.001). Regarding the goodness-of-fit indices, it indicated by GFI = 0.913, CFI = 0.939, IFI = 0.940, TLI = 0.926, and RMSEA = 0.063.

### 3.7. Convergent and Discriminant Validity

The value of AVE for both constructs exceeded 0.50, confirming the convergent validity of the AI-HSB scale. Moreover, the values of the square root of AVE exceeded the intercorrelation between the two constructs, which confirmed the discriminant validity of the AI-HSB scale (Table 3).

### 3.8. Reliability

In the CFA sample, the total number of items in the AI-HSB scale demonstrated an overall Cronbach’s alpha of 0.854. At the factor level, it was 0.91 for positive attitude toward AI-HSB and 0.67 for concern about using AI-HSB, respectively.

## 4. Discussion

In the present study, a scale called the AI-HSB has been developed and validated for measuring the level of reliance on AI tools among a university student in UAE for seeking psychological help. In all, it has been found that there are strong indications of factorial validity, reliability, and construct validity for the scale. The results support the idea of a multi-dimensional construct of AI-based help-seeking behavior, which covers positive attitude toward AI-HSB and use of AI for psychological distress.

After extensive investigation of the theories, models, scales, and experts, 13 items emerged, followed by a translation process of AI-HSB items into Arabic, and then both EFA and CFA were conducted, which yielded a two-factor solution as we hypothesized. EFA showed adequate sampling adequacy (KMO = 0.93) and a significant Bartlett’s test, confirming that the data were amenable to factor analysis. The two factors explained about 58% of the total variance, which is acceptable for newly constructed psychological measures (Smedslund et al., 2022). As we theoretically proposed, AI-HSB has two factors. The first factor, positive attitude toward AI-HSB, tapped students’ perceptions of AI resources as accessible, useful, confidential, and potentially stigma-reducing psychological support. The second factor, concern about using AI-HSB, tapped students’ doubts about the emotional authenticity, appropriateness, and need for AI-based psychological support. These dimensions support previous conceptualizations in TAM and barriers to help-seeking models, which suggest that technology-based help-seeking comprises empowering beliefs and perceived barriers. Our findings are consistent with a newly published paper which found two factors loading within five items to measure attitude toward AI tools for mental health support (Katsiroumpa et al., 2025).

The CFA also confirmed the two-factor structure with all items loading above recommended thresholds. The goodness-of-fit indices (GFI, CFI, IFI, TLI) of AI-HSB all exceeded 0.90, which was in parallel with literature (Abrahim et al., 2019). Although the RMSEA of 0.063 was slightly above the more stringent criterion of 0.05, it is still within the acceptable range for complex models in applied psychological research (Williams et al., 2022). The moderate correlation (r = 0.62) between the two factors suggests that, although related, positive attitudes and concerns are distinct constructs. This is further supported by the demonstration of convergent and discriminant validity, where AVE values exceeded 0.50 and square roots of AVE were greater than inter-factor correlations (Henseler et al., 2015).

The reliability indices were also satisfactory. The overall Cronbach’s alpha value of 0.854 reflects satisfactory internal consistency. The positive attitude scale has an excellent level of reliability with Cronbach’s alpha of 0.91; whereas, the concern scale has acceptable reliability with Cronbach’s alpha of 0.67, which is quite satisfactory given that it is a three-item scale (Bujang et al., 2018). Thus, we highly recommend refining and expanding the items of concern about AI-HSB to ensure proper internal consistency. The values of composite reliability further supported the soundness of the measurement model.

The finding of two dimensions is consistent with the current debates in the digital mental health literature, which highlight both the benefits and the ethical or emotional limitations of AI-based interventions. Students can appreciate the accessibility of AI tools while questioning the emotional validity and authenticity of these tools. This ambivalence is captured in the dual structure. Notably, AI-HSB seems to be a novel construct that goes beyond traditional help-seeking dispositions captured by conventional measures of professional or general help-seeking. Moreover, items of AI-HSB go beyond psychometrics, they may capture attitudes, intentions, perceived usefulness, or behavioral preferences simultaneously. However, the affordances offered by AI, such as anonymity, immediacy, perceived confidentiality, and lack of stigma, can potentially reduce psychological barriers to seeking help (Babu & Joseph, 2024). However, overdependence on AI, displacement of professional help, and lack of human contact are also potential concerns (Abdelwanis et al., 2026). The moderate correlation between positive attitudes and concerns indicates that students have complex and nuanced views about AI-mediated psychological help.

From the educational context in the UAE, where there is already a high level of integration of AI tools in academic environments, it is possible that the use of AI-HSB can be considered a culturally sensitive entry point for making psychological disclosures. Therefore, universities’ counseling centers should teach students the advantages and disadvantages of AI tools before seeking help. In contexts where there is a stigma associated with seeking help for mental health concerns, it is possible that AI-HSB can be used as a safer alternative. The perception of the stigmatizing effects of seeking help from AI is particularly interesting and should be further empirically explored. The use of AI-HSB tools should not be seen as a replacement for professional help. It should instead be seen as part of the help-seeking process. Students who are reluctant to seek help from therapists and psychiatrists may first seek help from AI-HSB. Theoretical models of help-seeking should include AI-HSB as a source of support.

This AI-HSB scale will allow universities, counselors, and policymakers to use a well-validated scale to measure the extent to which students are using AI for psychological coping. This will enable universities to monitor the use of AI for psychological help-seeking, identify students who are potentially relying on AI instead of seeking professional psychological help, develop educational strategies to encourage students to use AI in a balanced way, and inform their AI governance strategies in the context of higher education institutions. Given the widespread use of AI systems in universities, it is important to use evidence-based measures to monitor the use of AI systems. Without using well-validated measures, universities might underestimate the benefits and risks of AI-based psychological support.

However, several limitations are worth mentioning. Firstly, the study’s sample was collected by a convenience sample from a single institution in the UAE. However, this institution enrolls students from diverse nationalities and backgrounds. To overcome this, studies are needed to test measurement invariance in diverse nations, cultures, and types of institutions. Secondly, the study’s cross-sectional nature does not allow for the assessment of predictive validity, criterion-related validity, or stability over time. Future studies are needed to test test–retest reliability, examine the relationship between AI-HSB scale and other relevant scales, and predictive validity in terms of behavioral outcomes, such as changes in psychological well-being or professional help-seeking behaviors. Moreover, future studies are needed to examine relationships between AI-HSB and relevant constructs such as actual AI use, psychological wellbeing, mental health indicators, stigma, digital literacy, and traditional help-seeking behaviors. Thirdly, the concern dimension had only three items in the AI-HSB scale. Although reliability was satisfactory, future studies could refine and expand this dimension to include a greater range of concerns in the realms of ethics, emotions, and privacy concerns.

## 5. Conclusions

The current study proposes the AI-HSB as a valid and reliable tool for assessing AI-based psychological help-seeking behavior among a university student in the UAE. The proposed scale acknowledges the dual nature of the phenomenon, which is characterized by both endorsement and concern, emphasizing the complexity of the students’ attitude towards AI technology. In the wake of the increasing influence of AI on the educational and mental health environments, valid tools such as the AI-HSB are needed.

## Figures and Tables

**Table 1:** Demographic characteristics (*n* = 416).

Variables	Description	Frequency	Percentage %
Gender	Male	128	30.8%
	Female	288	69.2%
marital status	Single	389	93.5%
	Married	21	5%
	Widowed/divorced	6	1.5%
Age (year)	18–20	254	61.1%
	21–23	99	23.8%
	≥24	63	15.1%
Academic year	Freshman	205	49.3%
	Sophomore	96	23.1%
	Junior	72	17.3%
	Senior	43	10.3%
Major	Humanities and Sciences Fields	110	26.4%
	Business Fields	90	21.6%
	Medical Fields	94	22.6%
	Information Technology Fields	91	21.9%
	Architecture and art Fields	18	4.3%
	Engineering Fields	13	3.1%

**Table 2:** Factor loading of the AI-HSB scale (Varimax rotation) (*n* = 219).

#	Items	Factor 1	Factor 2
1	If I thought I was having a mental breakdown, my first thought would be to use an AI-based help-seeking tool (such as ChatGPT or a mental health chatbot).	0.78	
2	Talking about my problems with an AI-based system seems to me a good way to get rid of psychological problems.	0.85	
3	If I were experiencing a serious psychological crisis, I would be sure that an AI-based help-seeking tool would be useful.	0.80	
4	I admire people who are willing to cope with their problems, such as anxiety and stress, without seeking help from AI-based systems.		0.45
5	I want to get psychological support from an AI-based help-seeking tool if I am worried or depressed for a long period of time.	0.84	
6	I might want to use an AI-based help-seeking tool in the near future to solve my psychological problems.	0.81	
7	A person with a psychological problem prefers to ask for an AI help-seeking tool instead of going to a psychotherapist or psychiatrist.	0.57	
8	Given the amount of time and effort involved in using an AI-based help-seeking tool, I am sure that it would benefit someone like me.	0.80	
9	I believe that seeking psychological help from AI tools is the wrong choice because they are without feelings or emotions.		0.63
10	Anxiety, stress, and depression problems, like most things in life, tend to work out by themselves without the need for an AI-based help-seeking tool or psychiatrists.		0.74
11	If I had psychological problems and asked for AI seeking, it would keep it a secret.	0.55	
12	I believe that seeking psychological help from AI tools will reduce the stigma toward mental disorders.	0.66	
13	I recommend using AI tools as a quick and effective way to address my psychological problems.	0.82	
	Initial eigenvalues	6.181	1.354
	Percentages of variance explained	47.543	10.416
	Cumulative variance	47.543	57.959

**Table 3:** Cronbach’s alpha, CR, AVE, and bivariate correlations among the AI-HSB construct (*n* = 197).

#	Variable	1	2	Alpha	CR	AVE
1	Positive attitude toward AI-HSB	**0.720**		0.91	0.91	0.52
2	Concern about using AI-HSB	0.62	**0.730**	0.67	0.77	0.54

**Note:** CR: Composite reliability. AVE: Average variance extracted. Bold font numbers presented the square root of AVE.

## Data Availability

The data that support the findings of this study are available from the corresponding author upon reasonable request. The data are not publicly available due to privacy and ethical restrictions in accordance with Ajman University ethics committee policies.

## Supplementary Materials

The following supporting information can be downloaded at: https://www.mdpi.com/article/10.3390/ejihpe16070090/s1, Table S1: Development and Validation of AI Help-Seeking Behavior Scale Among Undergraduate University Students.

## Abbreviations

The following abbreviations are used in this manuscript:

AI	Artificial Intelligence
AI-HSB	Artificial Intelligence-help-seeking behavior
UAE	United Arab Emirates
