# Supplementary material for: Development and Validation of AI Help-Seeking Behavior Scale Among Undergraduate University Students
Source: Eur J Investig Health Psychol Educ. 2026 Jun 29;16(7):90. doi: 10.3390/ejihpe16070090 (PMC13408809; doi:10.3390/ejihpe16070090)
Supplement: Supplementary file 1 [file ejihpe-16-00090-s001.zip › ejihpe-4345575-supplementary-final version.pdf]

Development and Validation of AI Help-Seeking Behavior Scale Among Undergraduate University Students

Table S1. The items of AI-help seeking behavior in Arabic and English.

#	Items of AI	S. Agree	Agree	Disagree	S. Disagree
1	If I thought I was having a mental breakdown, my first thought would be to use an AI-based help seeking tool (such as ChatGPT or a mental health chatbot).<br>إذا مررتّ بانهيار نفسي، فسيخطر ببالي مباشرة هو استخدام أدوات الذكاء الاصطناعي مثل ChatGPT أو أحد برامج المحادثة المخصّصة للصحة النفسية.				
2	Talking about my problems with an AI-based system seems to me as a good way to get rid of psychological problems.<br>التحدث عن مشكلاتي النفسية مع أدوات الذكاء الاصطناعي هي طريقة فعّالة للتخلّص من مشكلاتي النفسية.				
3	If I were experiencing a serious psychological crisis, I would be sure that AI-based help seeking tool would be useful.<br>إذا مررت بأزمة نفسية شديدة، فسأكون متأكدًا من أن أدوات الذكاء الاصطناعي ستكوم مفيدة وستجد لي الحل.				
4	I admire people who are willing to cope with their problems such as anxiety and stress without seeking help from AI-based systems.<br>أنا أتعجب من الأشخاص الذين يستطيعون مواجهة مشكلاتهم كالقلق والتوتر والتكيف معها دون طلب المساعدة من أدوات الذكاء الاصطناعي.				
5	I want to get psychological support from an AI-based help seeking tool if I were worried or depressed for a long period of time.<br>أرغب بالحصول على الدعم النفسي من أدوات الذكاء الاصطناعي إذا كنتُ أشعر بالقلق والاكتئاب لفترة طويلة من الزمن.				

6	I might want to use an AI-based help seeking tool in the near future to solve my psychological problems.<br><br>قد أرغب في استخدام أدوات الذكاء الاصطناعي في المستقبل القريب لمعالجة مشكلاتي النفسية.				
7	A person with psychological problem is prefer to ask AI help seeking tool instead of going to psychotherapist and psychiatric.<br><br>إن الشخص الذي يعاني من مشكلة نفسية يفضل طلب المساعدة من أدوات الذكاء الاصطناعي بدل الذهاب إلى المعالج أو الطبيب النفسي.				
8	Given the amount of time and effort involved in using AI-based help seeking tool, I am sure that it would benefit someone like me.<br><br>أنا متأكد من أن أدوات الذكاء الاصطناعي ستستطيع حل مشكلاتي النفسي، نظرًا لكمية الوقت والجهد اللازمين لاستخدامها.				
9	I believe that seeking psychological help from AI tools is a wrong choice because it without feelings or emotions.<br><br>أعتقد أن الحصول على المساعدة النفسية من أدوات الذكاء الاصطناعي هو خيار خاطئ لأنه أداه بدون مشاعر أو أحاسيس.				
10	Anxiety, stress, and depression problems, like most things in life, tend to work out by themselves without the need for AI-based help seeking tool nor psychiatrics.<br><br>إن القلق والتوتر والاكتئاب، مثل معظم أمور الحياة، تُحلّ من تلقاء نفسها من دون الحاجة إلى أدوات الذكاء الاصطناعي أو الأطباء النفسيين.				
11	If I had psychological problems and ask AI-help seeking, it would keep it a secret.<br><br>أذا كان لدي مشاكل نفسية، أنا متأكد أن أدوات الذكاء الاصطناعي ستحفظ سري ولن تقولها لأحد				
12	I believe that seeking psychological help from AI tools will reduce the stigma toward mental disorders.				

	أعتقد أن طلب المساعدة النفسية من أدوات الذكاء الاصطناعي ستقلل من الاتجاهات السلبية تجاه الاضطرابات النفسية				
13	I recommend using AI tools as a quick and effective way to address my psychological problems.<br><br>أنصح باستخدام أدوات الذكاء الاصطناعي كأداة سريعة وفعالة لحل مشكلاتي النفسية				

We use A Four-point Likert-type scale from strongly agree to strongly disagree. The cutoff relies on low reliance on AI-HSB (1.00-1,99), moderate reliance on AI-HSB (2.00-2.99), and high reliance on AI-HSB (3.00-4.00). There are two constructs of AI-HSB. The first construct contained 10 items called (positive attitude toward AI-HSB). The second construct covered three items (items number 4, 9, and 10) labeled with concern about using AI-HSB and it should be in a reverse order.
